# Delta neutrophil index and symptomatic time are effective factors for predicting perforated appendicitis

**DOI:** 10.1097/MD.0000000000025935

**Published:** 2021-05-21

**Authors:** Min Jeong Kim, Won Hyuk Choi, Jin Cheol Cheong, Su Yun Choi, Jong Wan Kim, Jun Ho Park

**Affiliations:** a Department of Surgery, Kangdong Sacred Heart Hospital; b Department of Surgery, Dongtan Sacred Heart Hospital, Hallym University College of Medicine, Seoul, Republic of Korea.

**Keywords:** appendicitis, delta neutrophil index, perforation

## Abstract

Appendicitis is a common intra-abdominal inflammatory disease, and morbidity increases with age when perforation occurs. Because, not all patients require emergency surgery, there have been numerous studies on factors for predicting perforated appendicitis. In this study, we aimed to confirm whether the delta neutrophil index (DNI) and the time from symptom onset to surgery are effective predictors for perforated appendicitis in different age groups.

This was a retrospective study conducted on 542 appendicitis patients who underwent surgery at Kangdong Sacred Heart Hospital. The simple group consisted of 431 subjects, and the perforation group consisted of 111 subjects.

Multiple logistic regression analyses demonstrated that age, neutrophil percentage, DNI, C-reactive protein (CRP), and symptomatic time were significant predictors of perforation. Analysis of the receiver-operating characteristic curve showed that the DNI was the most reliable predictive value. In the analyses according to age, the perforation rate was higher in the >65-year-age group; these patients also had a higher DNI, CRP, and symptomatic time. In the DNI analysis using receiver operating characteristic (ROC) analysis, the area under the curve was higher in the >65-year-age group than in other age groups. In addition, the cutoff values have been determined and perforation occurred significantly in the group with a DNI value of 2.1 or higher and a symptomatic time of 33 hours or longer.

DNI is effective in predicting perforation in patients with appendicitis compared with other inflammatory factors. Furthermore, the simultaneous measurement of symptomatic time and DNI is helpful in predicting perforation and determining whether emergency surgery is necessary.

## 1 Introduction

Appendicitis is a common disease that frequently requires emergency surgery after the development of symptoms. In general, emergency surgery is performed to lower the incidence of complications or mortality that may occur as the inflammation progresses.^[1–3]^ Particularly, it has been reported that 50% to 70% of elderly appendicitis patients have perforation.^[4]^

However, the previously held belief that appendicitis always requires emergency surgery has changed in recent years. Indeed, several studies have reported that when antibiotics are administered at an early stage of diagnosis, semi-elective surgery can be performed to treat appendicitis.^[5–7]^ Semi-elective surgery is preferable since emergency surgery, which is often performed during the night, increases fatigue in surgeons and their assistants and consequently reduces their cognitive ability and judgment, thus becoming a risk factor for patient safety.^[8–10]^ Therefore, perforated appendicitis predictors have been studied in order to assist with decisions relating to whether emergency surgery or elective surgery should be performed.

There are biomarkers used as predictors of inflammation, including C-reactive protein (CRP), procalcitonin, elevated white blood cell (WBC) count, elevated serum bilirubin, and neutrophil-to-lymphocyte ratio (NLR), all of which have limitations.^[4]^ In recent years, studies on the delta neutrophil index (DNI), an automated hematology analyzer-based biomarker, have been published. DNI represents the fraction of immature granulocytes identified automatically by a cell analyzer machine.^[11]^ Immature immune cells enter the bloodstream during infection.^[12]^ The nuclear lobularity of white blood cells (WBC) and the cytochemical myeloperoxidase (MPO) reaction are useful predictive markers of infection by modern automated cell analyzers.^[13]^ The calculated value of the difference between the leukocyte differential in the MPO channel and the leukocyte differential in the nuclear lobularity channel in the DNI, which has been reported in previous studies as a factor related to disseminated intravascular coagulation scores, the positive blood culture, and mortality rate in sepsis patients.^[14]^ In addition, there have been several studies on the role of DNI as a predictor of perforated appendicitis in both young and old age groups. Despite this, to the best of our knowledge, no previous studies have compared each age group.

A recent study subdivided and analyzed the time from diagnosis of appendicitis to the time of surgery, and the time when the symptom occurred and the time after visiting the hospital; no correlation was found between perforation and the time from the hospital visit to the time of surgery.^[15]^

This study aimed to compare the effectiveness of DNI in predicting perforated appendicitis by age group and analyze the efficacy of DNI as a predictor by subdividing and analyzing the time from symptom onset to surgery.

## 2 Methods

### 2.1 Patients and characteristics

We retrospectively reviewed the medical records of all patients who were diagnosed with acute appendicitis and underwent appendectomy at Kang Dong Sacred-Heart Hospital for 2 years; a total of 542 patients were enrolled. This study was approved by the institutional review board of Kangdong Sacred Heart Hospital (Ref. 2016-10-022-001).

All data were fully anonymized before access, and the IRB waived the requirement for informed consent.

We excluded patients who underwent incidental appendectomy, interval appendectomy, negative appendectomy, or incision and drainage.

### 2.2 Data collection

The data collected relating to patient characteristics included age, sex, body mass index (BMI), WBC count, neutrophil percentage, CRP, DNI, and body temperature (BT) at initial diagnosis. The time from symptom onset to appendectomy was categorized into 3 periods. Symptomatic time is the period from symptom onset to hospital admission, hospitalization time is the period from admission to appendectomy, and the overall time is the period from symptom onset to appendectomy. We reviewed the electronic medical records of patients for time calculation. The timing of symptom onset was based on the patient's medical history at the first examination. If the time was not recorded, the closest 12 hours time was used as follows: if the first symptom occurred in the morning, the onset of symptoms was recorded as 6 am; if the first symptom occurred in the evening, the onset of symptoms was recorded as 6 pm; if the symptoms occurred 3 days before admission, the symptomatic period was recorded as 72 hours. Perforation was diagnosed based on the intraoperative findings and equivocal cases were diagnosed by pathologic reports.^[15]^

### 2.3 Laboratory tests and delta neutrophil index

Laboratory tests, which included DNI, WBC count, neutrophil percentage, and CRP, were measured before surgery. DNI is routinely recorded during complete blood count tests in our hospitals, and was determined using an automatic cell analyzer (ADIVA 2120 Hematology System; Siemens Healthcare Diagnostics, Forchheim, Germany). After red blood cell lysis, the cell size and stain intensity were measured using the tungsten-halogen-based optical system of the MPO channel to count and differentiate granulocytes, lymphocytes, and monocytes based on their size and MPO content. This was followed by cell counting and classification according to size, lobularity, and nuclear density, using the laser diode-based optical system of the lobularity nuclear density channel. DNI was calculated as the neutrophil and eosinophil subfraction measured in the MPO channel minus the polymorphonuclear neutrophil (PMN) subfraction measured in the nuclear lobularity channel, as previously described.^[16]^

### 2.4 Statistical analysis

All analyses were performed using SPSS 24.0 (IBM Corp., Armonk, NY). Continuous variables are presented as the mean ± SD and categorical variables as absolute numbers and percentages. Chi-square tests for categorical variables were used for categorical variables, and the independent-*t* test was used for continuous variables. We used binary logistic regression analysis to assess the perforation rate according to the WBC count, neutrophil percentage, CRP, fever, and DNI. Receiver-operating characteristic (ROC) curves were plotted and the Youden index method was used to determine the optimal cutoff values for DNI, WBC count, neutrophil percentage, and CRP level, for predicting perforation. To compare the diagnostic performance of each marker, the area under the curve (AUC) was calculated. *P*-value of <.005 was considered statistically significant in all analysis.^[16]^

## 3 Results

A total of 542 patients were enrolled in this study. The simple appendicitis group consisted of 431 patients and the perforated appendicitis group consisted of 111 patients. Table 1 shows a comparison of the characteristics of the 2 groups. The median age was significantly higher in the perforation group than in the simple group (43 vs 32 years old). The sex ratio was the same between the 2 groups (55.9% vs 44.1%, *P* = .991). The DNI, WBC count, neutrophil percentage, and CRP were significantly increased in the perforation group.

**Table 1:** Characteristics of patients according to the presence of perforation.

Characteristics	Simple (n = 431)	Perforation (n = 111)	
Age	32 (23–40)^∗^	43 (34–54)^∗^	<0.001
Sex			0.991
Male	241 (55.9%)	62 (55.9%)	
Female	190 (44.1%)	49 (44.1%)	
BMI, kg/m^2^	22.9 ± 4.14	23.6 ± 3.95	0.090
WBC, (×10^3^/μL)	11.7 ± 4.39	13.3 ± 5.02	0.001
Neutrophil percentage (%)	75.0 ± 11.54	83.0 ± 8.10	0.035
CRP, mg/dL	19.9 ± 7.23	46.6 ± 12.23	<0.001
BT, °C	36.9 ± 0.57	37.3 ± 0.86	0.112
DNI (%)	0.2 ± 4.83	3.4 ± 7.93	<0.001
Overall time	37.2 ± 34.40	45.4 ± 37.13	0.028
Symptomatic time	27.0 ± 32.55	34.7 ± 33.02	0.026
Hospitalization time	10.7 ± 9.98	11.8 ± 11.39	0.310

The overall time from symptom onset to appendectomy was significantly longer (by approximately 8.2 hours) in patients with perforated appendicitis than in patients with simple appendicitis (45.4 hours vs 37.2 hours, *P* = .028). The mean symptomatic time was 34.7 and 27.0 hours in patients with perforated appendicitis and patients with simple appendicitis, respectively, and this difference was statistically significant. However, the mean hospitalization time was not significantly different between the 2 groups.

Multivariate analysis showed that age, DNI, CRP, and symptomatic time were independent factors of appendiceal perforation. However, hospitalization time was not associated with perforation (Table 2).

**Table 2:** Multivariate logistic regression analysis of parameters for the prediction of perforated appendicitis.

Variable	Odds ratio	95% CI	*P*
Age	1.024	1.012–1.037	<.001
WBC	1.005	0.946–1.068	.867
Neutrophil percentage	1.102	1.012–1.037	.073
DNI	1.093	0.982–1.217	<.001
CRP	1.027	1.000–1.013	<.001
Overall time	0.999	0.983–1.016	.926
Symptomatic time	1.013	0.996–1.031	.036

**Figure 1:**
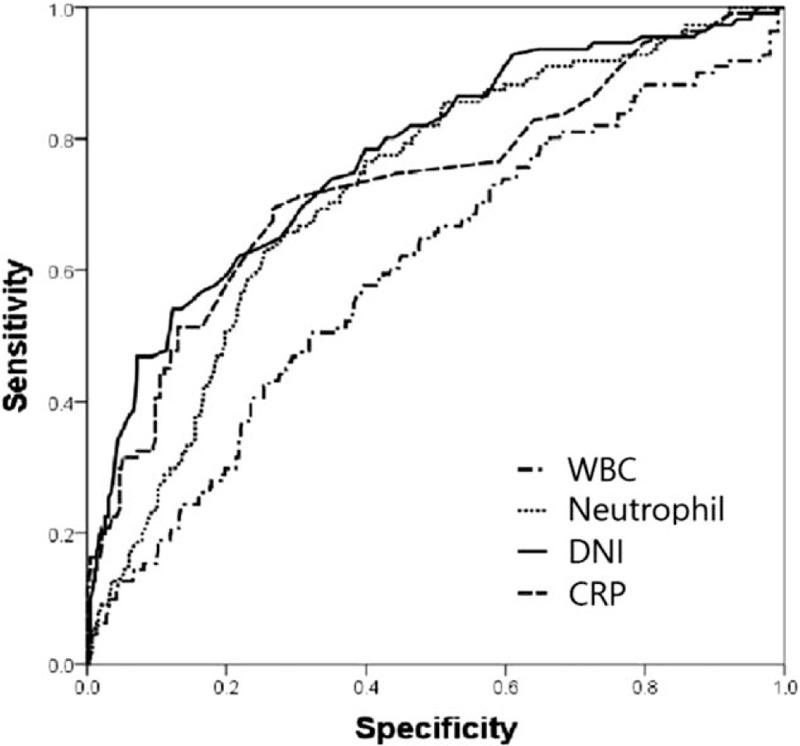
Receiver-operating characteristic curves of predictors associated with perforation.

**Figure 2:**
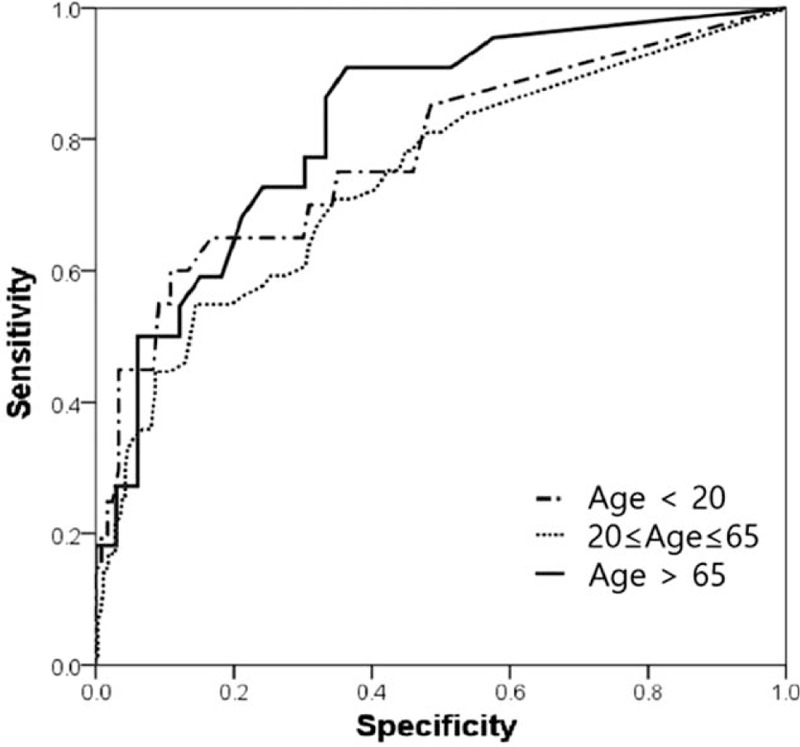
Receiver-operating characteristic curves of DNI by age group. DNI = Delta neutrophil count.

In the ROC curve shown in Fig. 1, the AUC of DNI was 0.773, which was higher than that of the WBC, neutrophil percentage, and CRP (Table 3). As shown in Fig. 2, the AUC was 0.823 in the >65-year-age group, which was higher than that in the other groups (0.787 and 0.739). Thus, the ROC curve revealed that the DNI of the >65-year-age group showed better performance for perforated appendicitis (Table 4). In other words, DNI was a more effective predictor of perforated appendicitis in the older age group than in other age groups.

**Table 3:** Values of laboratory markers for differentiating between simple and perforated appendicitis by receiver characteristic curve.

Variable	AUC (95% CI)	Cut-off value	Sensitivity (%)	Specificity (%)
DNI	0.773 (0.722–0.824)	2.1	67.6	89.1
WBC	0.599 (0.538–0.660)	13.8	50.5	68
Neutrophil percentage	0.723 (0.671–0.774)	83.4	63.1	74.5
CRP	0.771 (0.704–0.818)	16.7	75.7	74.0

**Table 4:** AUCs and 95% CIs of DNI by age group.

Variable	AUC (95% CI)
Age < 20	0.764 (0.712–0.817)
20 ≤ Age ≤ 65	0.739 (0.669–0.808)
Age > 65	0.823 (0.711–0.935)

**Figure 3:**
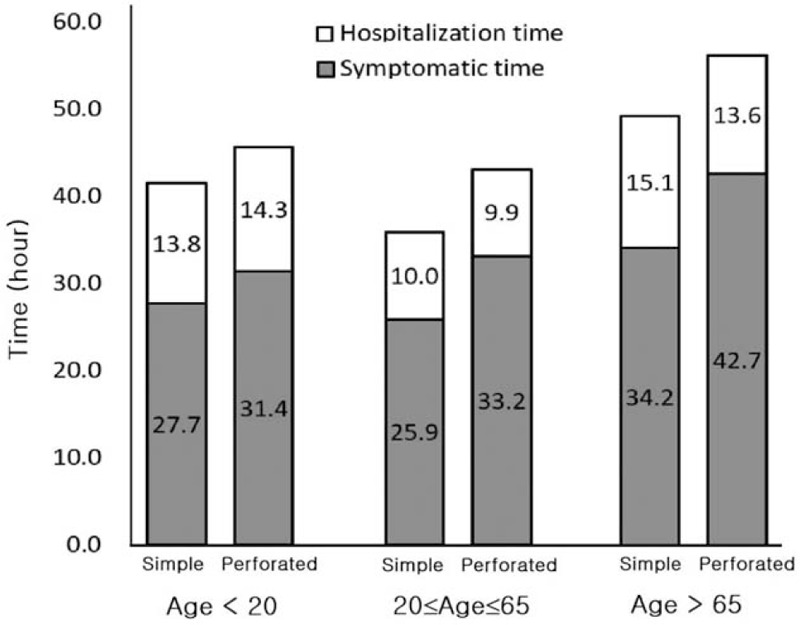
Comparison of hospitalization and symptomatic time between simple appendicitis and perforated group according to age group.

In terms of the characteristics according to age, the perforation ratio was 31.9% in the >65-year-age group, which was higher than that in the other groups; the CRP and DNI were also significantly higher in the older age group. In terms of time factors, the overall time, symptomatic time, and hospitalization time were significantly higher in the old age group (Table 5). In Fig. 3, the hospitalization time in each age group was not significantly different between the simple appendicitis group and the perforated appendicitis group, but there was a difference in symptomatic time.

**Table 5:** Characteristics of patients according to age group.

Characteristics	Age < 20 (n = 140)	20 ≤ Age ≤ 65 (n = 308)	Age > 65 (n = 94)	*P*
Perforation. ratio	20 (14.3%)	61 (19.8%)	30 (31.9%)	<.001
WBC	12.86	11.89	10.79	.11
Neutrophil count	76.1	76.7	77.8	.623
CRP	5.0	6.2	20.4	.013
DNI	0.9	1.6	2.6	.017
Overall time	38.7	36.9	51.2	.012
Symptomatic time	28.2	27.3	37.6	.004
Hospitalization time	11.9	10.0	14.53	.016

The optimal cutoff DNI for predicting perforation was 2.1, with a sensitivity of 67.6% and specificity of 89.1%. The optimal cutoff symptomatic time for predicting perforation was 33 hours, with a sensitivity of 47.7% and specificity of 77.8%; the cutoff value was calculated using the Youden index. DNI with symptomatic time showed a specificity of 97.0%, and elevated specificity was presented than DNI or symptomatic time alone (Table 6). This result indicates that when patients with appendicitis arrive at the hospital, the DNI is above 2.1% and symptomatic time is longer than 33 hours, the rate of perforated appendicitis is 97%, and emergency operation will be planned.

**Table 6:** Performance of delta neutrophil index and symptomatic time.

	Cut-off level	Sensitivity (%)(%)	Specificity (%)	Positive predictive value (%)	Negative predictive value (%)
DNI	2.1%	67.6%	89.1%	61.5%	91.4%
Symptomatic time	33 hr	47.7%	77.8%	35.6%	85.2%
DNI + Symptomatic time		32.4%	97.0%	73.5%	84.8%

## 4 Discussion

The present study revealed that DNI is a reliable predictor of perforated appendicitis. Among the WBC count, neutrophil percentage, CRP, and DNI, DNI was the most reliable predictor, with an AUC value of 0.773. Several studies have analyzed the relationship between serological markers and the severity of appendicitis. For example, Qi and Zhang^[17]^ reported that the neutrophil percentage and CRP were risk factors for gangrenous appendicitis based on logistic regression analysis. In addition, Xharra et al^[18]^ analyzed the relationship using a variety of biomarkers, including CRP, WBC count, and neutrophil percentage, and reported that these markers increased with the severity of inflammation. In recent years, many studies on DNI as an inflammatory biomarker have been carried out; these include a study on the role of DNI as a predictor of mortality in sepsis patients, and a study on the role of DNI as a predictor of perforated appendicitis.^[4,16,19]^ Shin et al^[4]^ recently reported that the perforated appendicitis rate was as high as 36% and the cutoff value of DNI was 1.4 in the >65-year-age group. Kim et al^[19]^ reported that DNI is a reliable predictive value for complicated appendicitis in children. However, to the best of our knowledge, the current study is the first to compare DNI between age groups. In the present study, perforated appendicitis was more common and the mean value of DNI was also significantly higher in the older age group. In particular, the AUC value of DNI was higher than that in the other age groups, indicating that DNI plays a more significant role in predicting perforated appendicitis in the >65-year-age group than in other age groups.

Serum CRP is widely used as an objective index of disease activity and a plasma protein, and its concentration increases dramatically as a result of cytokine-mediated responses to most forms of tissue injury, infection, and inflammation.^[17]^ In this study, CRP was also a reliable predictor of perforated appendicitis. However, DNI does not require any additional time or cost in clinical settings, unlike other biomarkers such as CRP and procalcitonin, and is increasingly being performed as a routine laboratory test, along with complete blood count.

The present study revealed that the overall time from onset of symptoms to appendectomy was significantly longer in patients with perforated appendicitis than in patients with simple appendicitis. When the duration of symptoms was divided into the symptomatic period before admission and the hospitalization period, symptomatic time was significantly associated with the rates of perforated appendicitis.

However, there is controversy regarding the association between the symptomatic period and hospitalization period with complications. A previous study showed that increased hospitalization time increased the risk of perforated appendicitis, although this study was only based on 24 hours.^[20,21]^ Furthermore, Eldar et al^[22]^ reported that delayed admission was associated with an increased rate of complications related to infection (*P* < .001) and advanced appendicitis (*P* < .001); however, hospital delay (termed “physician delay” in the study) was not associated with the stage of disease. Maroju et al^[23]^ reported that postoperative complications for acute appendicitis were associated with a delay in treatment, which was not due to in-hospital delay (with vs without complications: 8.6 hours vs 8.3 hours, *P* = not significant) but rather patient delay (63.3 hours vs 24.3 hours, *P* < .001). In addition, this study reported that the mean hospitalization time was 10.9 hours, and there were only a few cases with a hospitalization time >24 hours, thus revealing that hospitalization did not affect the risk of perforated appendicitis.

The analysis according to age showed that the rate of perforated appendicitis was higher in the >65-year-age group, and that the symptomatic time and hospitalization time were significantly longer. Previous studies have suggested that a higher proportion of elderly patients than younger patients present with perforated appendicitis.^[24]^ One possible explanation is that elderly patients sometimes present with ambiguous features and fewer elderly patients report right lower quadrant pain.^[25]^ These atypical symptoms may make accurate diagnosis difficult. Another possible explanation relates to physiologic changes in elderly patients, including decreased immunity.^[26]^ The hospitalization time was also longer in the older age group than in the other groups since it takes longer to prepare preoperative management and fully evaluate the patient's cardiac pulmonary and renal function.

DNI and symptomatic time are significant predictive factors of perforated appendicitis. Over the cutoff value, DNI had a specificity of 89.1%, while the symptomatic time had a specificity of 77.8%. However, together, these 2 factors show a high specificity of 97.0%; these results are higher than the specificity of CRP (72%) in the study by Xharra et al.^[17]^ Therefore, DNI and symptomatic time are useful indicators of perforated appendicitis and may also be helpful in deciding whether emergency surgery is necessary.

There are several limitations to this study. This was a retrospective review of the medical records. For this reason, we could only evaluate data for the WBC count, neutrophil percentage, CRP, and those that were routinely measured at the hospital. Other inflammatory markers, such as procalcitonin and lactic acid, are not routinely assessed and therefore could not be analyzed in this study. Because of the small sample size in this study, additional studies with larger numbers of patients are required to validate the clinical usefulness of DNI as a predictive factor of perforation in appendicitis.

Despite these limitations, our results demonstrate that DNI is a useful marker for predicting perforation in appendicitis, and that the time from symptom onset to surgery is associated with the risk of perforation.

## 5 Conclusion

The present study showed that DNI was more accurate than the WBC count, neutrophil percentage, and CRP for predicting perforation in appendicitis patients. In addition, the time analysis combined showed that the time taken after symptom onset affected perforation but time after hospital visit was not associated with perforation. Moreover, a DNI > 2.1 at 33 hours after symptom onset was a strong predictor of perforation.

## Acknowledgments

The authors thank Yun mi Kim and Seong man Cha for their valuable support of this research.

## Author contributions

**Conceptualization:** Min Jeong Kim, Jong Wan Kim, Jun Ho Park.

**Data curation:** Min Jeong Kim, Su Yun Choi, Jong Wan Kim, Jun Ho Park.

**Formal analysis:** Min Jeong Kim.

**Methodology:** Min Jeong Kim, Jong Wan Kim.

**Supervision:** Jun Ho Park.

**Visualization:** Min Jeong Kim.

**Writing – original draft:** Min Jeong Kim.

**Writing – review & editing:** Min Jeong Kim, Won Hyuk Choi, Jin Cheol Cheong, Jun Ho Park.
